# Near-complete telomere-to-telomere genome assembly of a male barbel steed (*Hemibarbus labeo*)

**DOI:** 10.1038/s41597-026-07825-9

**Published:** 2026-07-06

**Authors:** Wei Liu, Ming Qi, Wenrui Shi, Xiaofeng Zhang, Gaohua Yao, Qinghui Meng, Chen Chen, Tianlun Zheng, Yongyi Jia

**Affiliations:** Zhejiang Fisheries Technical Extension Center, Hangzhou, 310023 China

## Abstract

The barbel steed (*Hemibarbus labeo*), an economically important freshwater fish in China, belongs to the order Cypriniformes and the family Cyprinidae. At present, research has been conducted on the basic biology, artificial breeding, aquaculture, and nutritional requirements of *H. labeo*. However, genome assembly and exploration of the unique genomic composition of the barbel steed are still in their infancy. Previously available genome assemblies were fragmented and incomplete, hindering advances in genomic research and breeding applications. In this study, we generated a near complete telomere-to-telomere (T2T) genome assemblies for a male *H. labeo* using PacBio HiFi, ONT ultra-long, and Hi-C data. The resulting assemblies span 1,040.46 Mb with contig N50 of 41.15 Mb, and 25 chromosomes were assembled. The quality value (QV) scores were 60.02, and Benchmarking Universal Single-Copy Orthologs (BUSCO) completeness scores were 98.3%. The genome contains a total of 37,023 coding genes, of which 35,589 (96.13%) were functionally annotated. The high-quality near-T2T assemblies provide invaluable resources for molecular breeding and advancing biological and evolutionary research in *H. labeo*.

## Background & Summary

Barbel steed (*Hemibarbus labeo*) (Fig. 1) is a freshwater bottom dwellers cyprinid living in streams and feeding on aquatic insects and mollusks, and also ingest algae, plant detritus, small shrimp, and smaller fish. Distributed in major drainage of East Mainland China, Japan, and Korea. It has both ecological and economic value and is an important target for the conservation of river fish diversity. It belongs to the family Hemibarbus, the body is laterally compressed and rod-shaped, with a large head, pointed snout and rounded abdomen. *H. labeo* has often been misidentified as its closely related sister species, *H. maculatus*. Adult *H. labeo* have no spots on their sides, only small individuals have inconspicuous black spots, while *H. maculatus* have 8–14 round dark brown spots on their body sides^1^. Because of *H. labeo* are tender and delicate, with a delicious flavor, the content of unsaturated fatty acid DHA reaches the level found in deep-sea fish, offering high nutritional value and health benefits, the species has been an important farmed fish in China^2–4^.

**Fig. 1:**
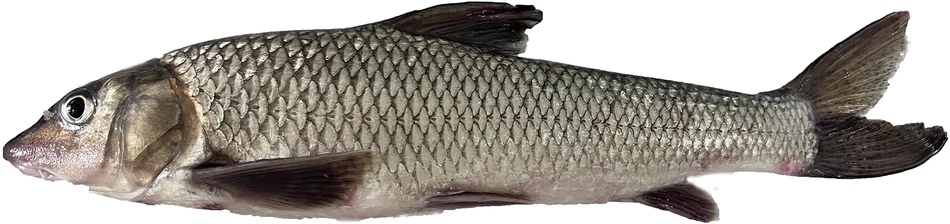
Barbel steed, *H. labeo* collected from Hangzhou, Zhejiang Province, China.

At present, several genome assemblies of *H. labeo* have been previously reported, but they are all limited to the chromosome level and contain numerous gaps and incomplete telomeric sequences^5,6^. Incomplete genome data is like a ‘treasure map’ with missing key areas, which can seriously mislead or even hinder the progress of scientific research. In recent years, the rapid development of high-throughput sequencing technology and their gradual reduction in costs has made large-scale genome sequencing and assembly feasible in non-model taxa^7–9^. In this study, we used a combination of PacBio HiFi, ONT ultralong, Hi-C reads and RNA-Seq sequencing data to assemble and annotate the *H. labeo* genome. The results revealed an assembled genome size of 1,040.46 Mb with 25 chromosomes were assembled. The completeness of the genome assembly was assessed using a number of parameters, which include scaffold N50 score (41.15 Mbp), BUSCO score (97.5%), QV score 60.02. In addition, a total of 22,518 high-confidence protein-coding gene loci were identified, among which 20,143 (89.5%) were successfully annotated in public functional databases. The assembled chromosome-level genome offers invaluable resources for molecular breeding, functional genomics, and evolutionary studies of *H. labeo* and other Cyprinidae species.

## Methods

### Ethics statement

The experimental procedures involving animals were approved by the Institutional Review Board of the Science Technology Department of Zhejiang Province for Zhejiang Fisheries Test and Aquatic Disease Prevention Center [SYXK (Zhe) 2025–0015, 13 Jun 2025].

### Sampling and sequencing

A sexually mature male individual of *H. labeo* was collected from Hangzhou, Zhejiang Province, China. Tissue specimens, including muscle, liver, and intestine, were dissected and immediately frozen in liquid nitrogen for nucleic acid extraction. Genomic DNA was isolated from muscle using the Blood & Cell Culture DNA Kit (Qiagen, 13323). DNA quality was evaluated via NanoDrop (Thermo Fischer Scientific, ND2000), Qubit(Invitrogen, Q33238), and agarose gel electrophoresis. Total RNA was extracted from six tissue types using the RNAprep Pure Tissue Kit (TIANGEN, DP431). RNA integrity, concentration, and purity were assessed using the NanoDrop (Thermo Fischer Scientific, ND2000) and Angilent 2100 Bioanalyzer instrument (Agilent Technologies, G2939BA).

### Library construction, and sequencing

A multi-platform sequencing strategy was adopted for genome analysis of *H. labeo*.

For PacBio HiFi sequencing, a SMRT bell library was constructed using a SMRTbell Express Template Prep Kit 2.0 (Pacific Biosciences, CA, USA). The library preparation process involved DNA shearing, purification with AMPure PB Beads, removal of single-stranded DNA overhangs, damage repair, end repair, ligation of hairpin adapters, and final bead-based purification. After passing quality control, the library was loaded onto a PacBio platform (Pacific Biosciences, CA, USA). HiFi reads were generated using SMRTLink software (https://www.pacb.com/support/software-downloads). The final dataset consisted of 2,370,484 HiFi reads, totaling 40.36 Gb of sequence data, with an average read length of 17.03 kb and an N50 of 16.58 kb (Table 1).

**Table 1:** Sequencing data generated for *H. labeo* genome assembly.

Library type	Platform	Library size	Data size (Gb)	Average depth(x)	Average Length
Ultra-long reads	Oxford Nanopore PromethION	—	71.88	71	61.50 kb
HiFi reads	PacBio Revio	10~20 Kb	40.36	40	17.03 kb
Hi-C	DNBSEQ-T7	350 bp	119.34	115	150 bp

For ONT ultra-long sequencing, libraries were prepared with the SQK-LSK109 Kit (Oxford Nanopore Technologies, UK) and sequenced on Nanopore PromethION sequencer. Raw data were processed using Porechop (version 0.2.4, https://github.com/rrwick/Porechop) to remove adapter sequences, followed by Filtlong (version 0.2.1, https://github.com/rrwick/Filtlong) with parameters “-min_length 30000–min_mean_q 90” to filter out low-quality reads. This yielded 71.88 Gb of high-quality ultra-long reads with an average length of 61.50 kb (Table 1).

The Hi-C library were generated through the following steps: crosslinking cells with formaldehyde, digestion with the restriction enzyme MboI, end-filling and biotin labeling, ligation of blunt-ended fragments, purification, and random shearing to 300–500 bp fragments^10,11^. Library quality was checked using a Qubit 2.0 fluorometer, an Agilent 2100 instrument (Agilent Technologies, CA, USA), and q-PCR. Paired-end sequencing (150 bp) was subsequently performed on an Illumina Novaseq platform. To eliminate reads with artificial bias (e.g., low-quality paired reads resulting from base-calling errors, duplicates, or adapter contamination), we filtered out reads containing >3 unidentified nucleotides (N), reads aligning to adapters, and reads with ≥20% of bases having a Phred quality score ≤5. A total of 397,797,464 raw reads were generated, resulting in 119.20 Gb of clean data for pseudochromosome assembly (Table 1).

### Genome assembly

Whole-genome assembly was performed using Hifiasm (version 0.25.0, https://github.com/chhylp123/hifiasm)^12^, integrating HiFi, ONT, and Hi-C data. The initial ONT assembly was polished by PacBio HiFi reads using Racon (version 1.4.3, https://github.com/isovic/racon)^13^. The draft assembly was then processed with purge_dups (version 1.2.5, https://github.com/dfguan/purgedups)^14^ to remove haplotigs and overlaps based on read depth. Remaining contigs were aligned against the NT database using BLAST (version 2.12.0, https://blast.ncbi.nlm.nih.gov/Blast.cgi)^15^ to identify and remove potential contaminant sequences. A GC-depth plot was used to filter out contigs exhibiting unusually high GC content or low coverage. The preliminary assembly achieved a genome size of 1.06 Gb, consisting of 29 contigs with a contig N50 of 40.80 Mb (Table 2).

**Table 2:** Preliminary genome assembly results.

Term	Before filtering	After filtering
Contig number	60	29
Contig length(bp)	1,063,336,482	1,061,389,113
GC content(%)	38.62	38.59
Contig Min length(bp)	16,084	593,083
Contig N50(bp)	40,804,088	40,804,088
Contig N90(bp)	31,873,865	31,873,865
Contig Max(bp)	58,618,877	58,618,877

Filtered Hi-C reads were aligned to the initial assembly using HiC-Pro (version 3.1.0, https://github.com/nservant/HiC-Pro)^16^. Valid paired-end reads were used for pseudochromosome construction with 3D-DNA (180922, https://github.com/theaidenlab/3d-dna)^17^. Manual correction of pseudochromosomes was carried out in Juicebox, guided by Hi-C contact maps. The final contact map was visualized using HiCExplorer (version 3.7.2, https://hicexplorer.readthedocs.io/en/latest/)^18,19^. To obtain a telomere-to-telomere (T2T) gap-free genome, we applied a custom gap-filling pipeline using Auto Gap Filling software. Gap filling was performed based on synteny analysis between the supplemental genome and the target genome. For each gap, a sliding window (step size: 10 kb) was used to extract 10–100 kb upstream and downstream flanking sequences. Syntenic relationships were identified by aligning these flanking regions to the supplemental genome. Subsequently, the target chromosome and the corresponding candidate contig from the supplemental genome were aligned using MUMmer for each gap to determine the exact filling position. Based on the alignment-derived positional information, the genome was reconstructed to obtain a gap-filled assembly. Contigs from NextDenovo (version 2.5.2, https://github.com/Nextomics/NextDenovo) were aligned to Hifiasm-derived contigs using Winnowmap2 (version 2.03, https://github.com/marbl/Winnowmap)^20^. ONT contigs located near or spanning gaps were selected for gap filling at the chromosome level. These selected ONT contigs were aligned to the corresponding chromosome sequences using MUMmer (version 4.0.0, https://github.com/mummer4/mummer)^21^, and gap sequences were filled based on the ONT contig alignments. The final assembly contained 25 chromosomes with a scaffold N50 of 41.15 Mb. Although not all chromosomes have telomeric repeats at both ends, all of them are gap-free, achieving near T2T completeness (Fig. 2; Tables 3, 4). The 25 chromosomes were clearly distinguishable in the heatmap (Fig. 3), and the strong interaction signals along the diagonal further confirmed the high quality of the assembly.

**Fig. 2:**
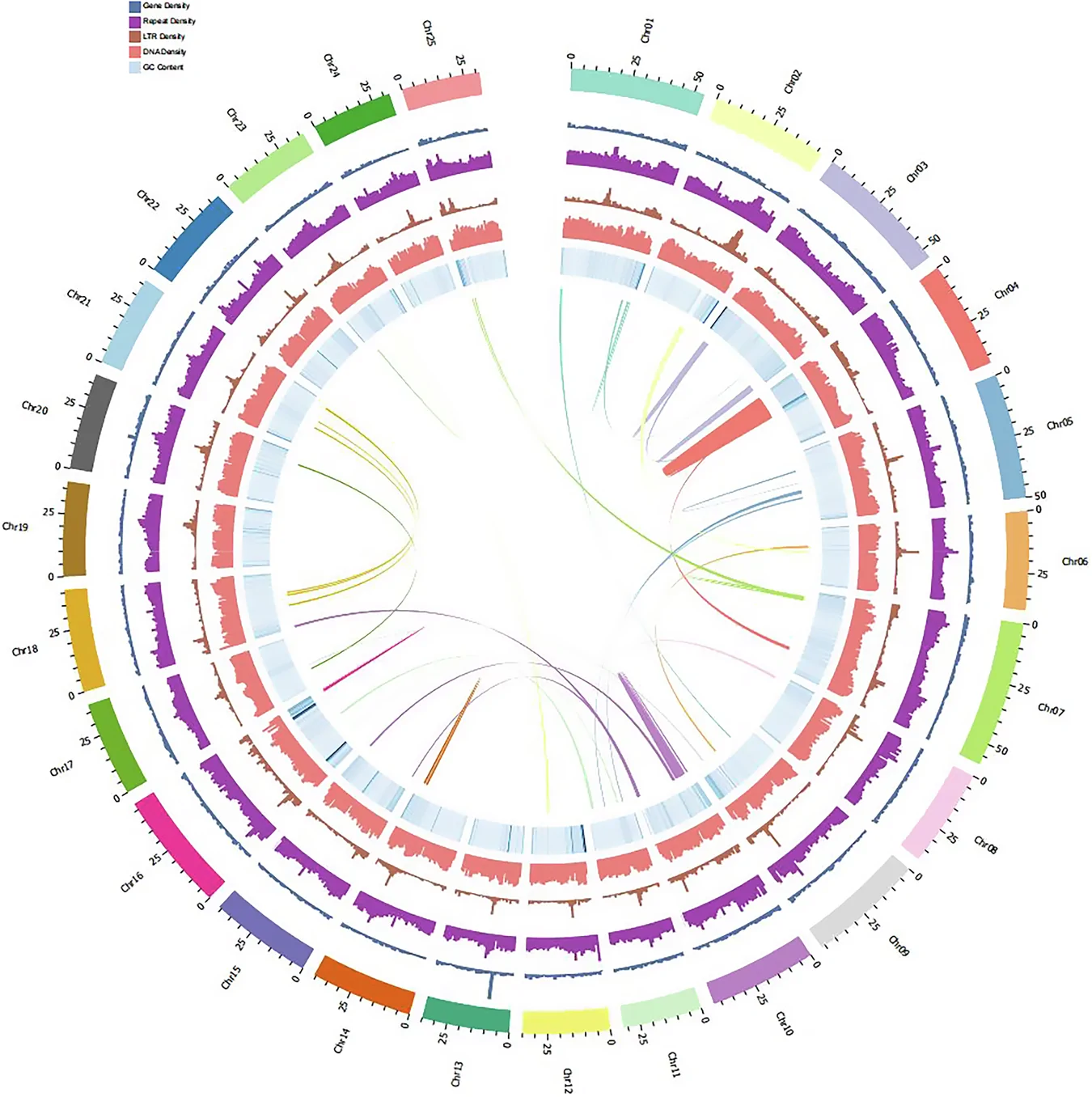
Circos plot of the distribution of the genomic elements in *H. labeo*. Tracks from outer to inner rings represent: gene density (blue), repeat density (purple), LTR element density (brown), DNA transposon density (red), and GC content (turquoise).

**Table 3:** Final genome assembly statistics of *H. labeo*.

Hi-C		ONT		Gap filling	
Scaffold number	31	Contig Num	35	Scaffold number	25
Scaffold length(bp)	1,061,390,113	Contig Len(bp)	1,135,646,304	Scaffold length(bp)	1,040,455,555
GC content (%)	38.59	GC content (%)	38.74	GC content (%)	38.59
Contig N50(bp)	40,804,088	Contig Min(bp)	56,133	Contig N50(bp)	41,151,637
Scaffold N50(bp)	40,804,088	Contig N50(bp)	42,713,096	Scaffold N50(bp)	41,151,637
Chromosome percent(%)	97.99	Contig Max(bp)	56,666,158	Chromosome percent(%)	100.00

**Table 4:** Statistics of chromosome lengths and gap number in *H. labeo*.

Chromosome	Gap Numbe	Chromosome Length(bp)	Chromosome	Gap Numbe	Chromosome Length(bp)
chr1	0	58,618,877	chr14	0	39,618,306
chr2	0	54,048,119	chr15	0	38,843,819
chr3	0	53,998,202	chr16	0	38,748,387
chr4	0	50,155,832	chr17	0	38,202,825
chr5	0	47,831,082	chr18	0	37,866,293
chr6	0	46,979,734	chr19	0	37,030,990
chr7	0	46,601,314	chr20	0	36,544,834
chr8	0	43,308,892	chr21	0	35,077,139
chr9	0	43,105,827	chr22	0	35,045,071
chr10	0	41,303,597	chr23	0	32,480,683
chr11	0	41,151,637	chr24	0	31,873,865
chr12	0	40,804,088	chr25	0	31,568,348
chr13	0	39,647,794

**Fig. 3:**
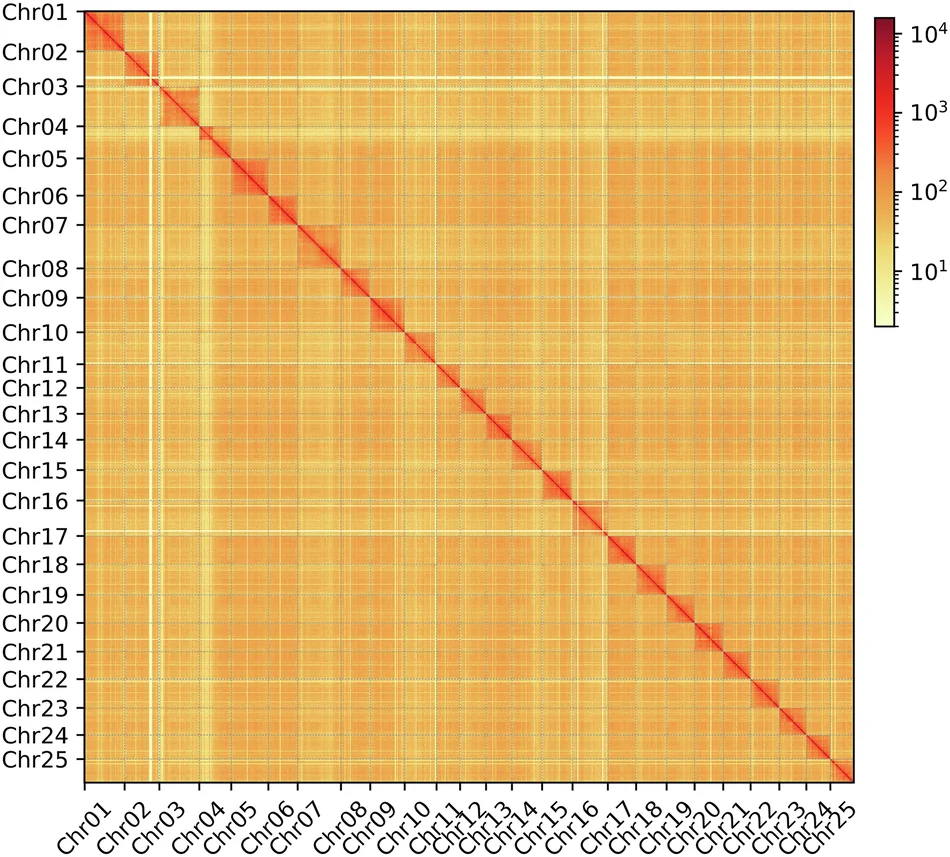
A heatmap of chromosome interaction in *H. labeo*. The blocks represent the 25 pseudochromosomes showing, clear chromosomescale scaffolding and interaction signals.

### Telomere evaluation, extension, and centromere analysis

Following gap filling, telomeric and centromeric regions were identified. Telomere analysis was performed by scanning the genome for the telomeric repeat motif “TTAGGG”. For chromosomes lacking telomeres or having excessively short telomeric regions, we extended them sequentially using ONT assembly sequences and HiFi reads, followed by re-evaluation. Obtain the specific positional distribution of telomeres on chromosomes (Table 5). Centromeric regions were characterized by identifying continuous clusters with high densities of short tandem repeats and low gene densities. Centromics(https://github.com/ShuaiNIEgithub/Centromics) software was used for centromere prediction based on HiFi reads and the final assembled genome. For some chromosomes, no centromeric signals were detected. These instances may correspond to non-canonical satellite repeat regions often containing complex insertions of multiple satellite families, long terminal repeat (LTR) retrotransposons, or other uncharacterized transposable elements that fall beyond the detection capability of tandem repeat–based tools. Presumed centromeres were successfully identified on 20 chromosomes, whereas five chromosomes lacked definitive centromeric signals (Fig. 4; Table 6).

**Table 5:** Statistical distribution of telomeres on each chromosome of the genome in *H. labeo*.

Chromosome	Left start	Left end	Left length	Right start	Right end	Right length
chr1	1,202	26,816	25,615	—	—	—
chr2	93	5,070	4,978	54,008,867	54,032,848	23,982
chr3	297	3,523	3,227	—	—	—
chr4	60,360	79,334	18,975	50,150,080	50,155,823	5,744
chr5	1,034	35,476	34,443	47,825,802	47,831,048	5,247
chr6	147	1,116	970	46,969,135	46,979,729	10,595
chr7	—	—	—	46,599,632	46,601,298	1,667
chr8	—	—	—	43,292,541	43,307,361	14,821
chr9	31,683	60,454	28,772	—	—	—
chr10	—	—	—	41,231,170	41,303,002	71,833
chr11	—	—	—	—	—	—
chr12	—	—	—	40,758,137	40,792,422	34,286
chr13	48	2,600	2,553	39,632,368	39,635,807	3,440
chr14	3	7,879	7,877	—	—	—
chr15	—	—	—	38,841,059	38,843,815	2,757
chr16	38,307	38,515	209	38,743,175	38,748,383	5,209
chr17	450	19,771	19,322	38,200,410	38,201,499	1,090
chr18	2,881	11,160	8,280	37,824,394	37,831,396	7,003
chr19	1,913	62,638	60,726	37,025,966	37,030,063	4,098
chr20	5	4,769	4,765	36,478,838	36,522,281	43,444
chr21	605	27,257	26,653	—	—	—
chr22	15,493	85,211	69,719	35,037,488	35,044,534	7,047
chr23	—	—	—	32,471,999	32,480,678	8,680
chr24	2,378	33,053	30,676	31,792,734	31,873,865	81,132
chr25	371	20,086	19,716	—	—	—

**Fig. 4:**
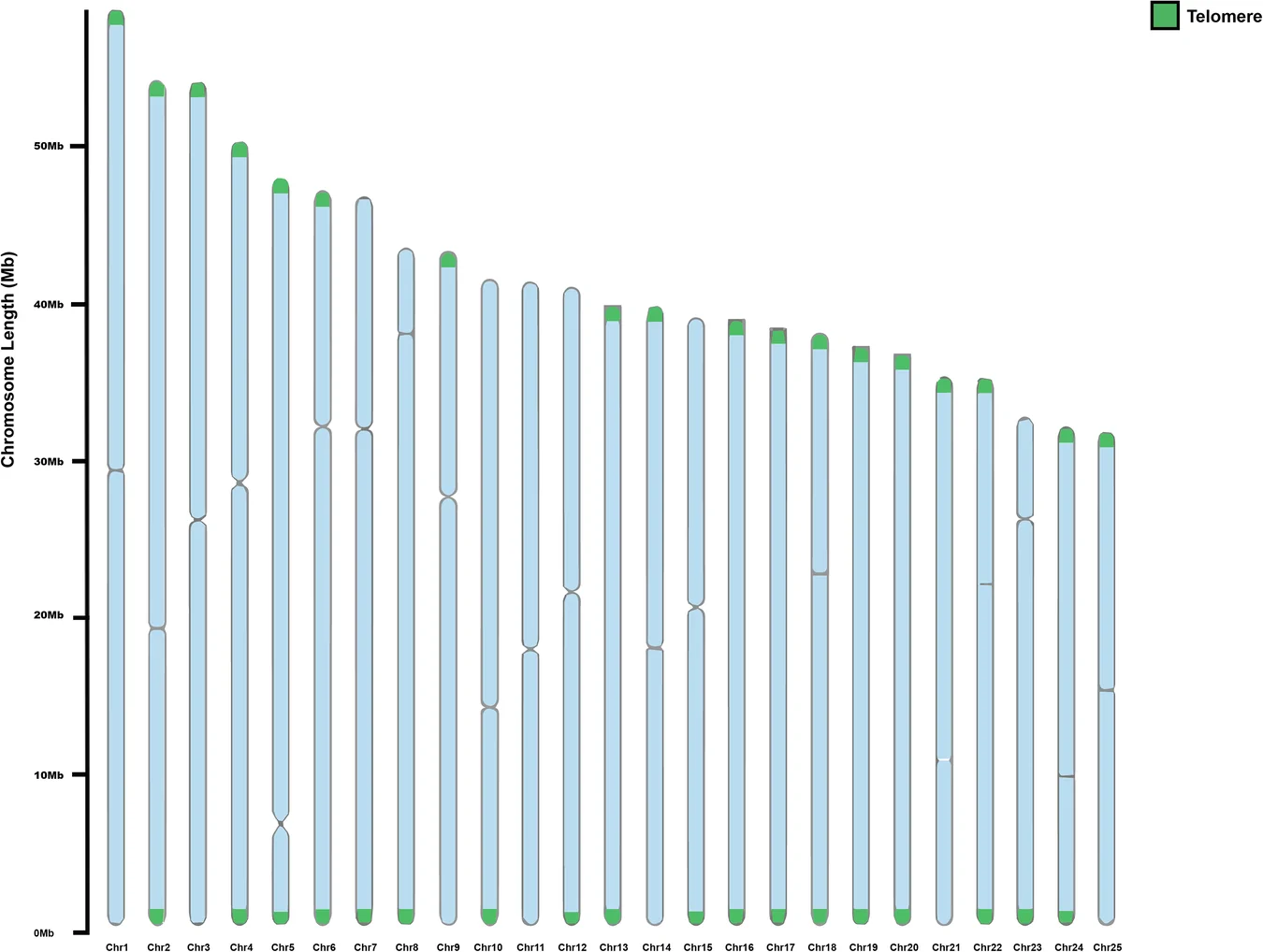
Presentation of telomeres and centromeres of Telomere-to-Telomere genome assemblies of *H. labeo*.

**Table 6:** Statistical distribution of centromeres on each chromosome of the genome in *H. labeo*.

Chromosome	Chrromosome length	Centromer start	Centromer end	Centromer length
chr1	58,618,877	29,380,000	29,640,000	260,001
chr2	54,048,119	34,920,000	35,200,000	280,001
chr3	53,998,202	27,780,000	28,340,000	560,001
chr4	50,155,832	21,390,000	22,280,000	890,001
chr5	47,831,082	40,700,000	42,030,000	1,330,001
chr6	46,979,734	14,780,000	15,350,000	570,001
chr7	46,601,314	14,580,000	15,090,000	510,001
chr8	43,308,892	5,360,000	5,550,000	190,001
chr9	43,105,827	15,380,000	16,000,000	620,001
chr10	41,303,597	27,160,000	27,570,000	410,001
chr11	41,151,637	23,190,000	23,800,000	610,001
chr12	40,804,088	19,190,000	19,770,000	580,001
chr13	39,647,794	—	—	—
chr14	39,618,306	21,720,000	22,010,000	290,001
chr15	38,843,819	18,190,000	18,800,000	610,001
chr16	38,748,387	—	—	—
chr17	38,202,825	—	—	—
chr18	37,866,293	15,280,000	15,460,000	180,001
chr19	37,030,990	—	—	—
chr20	36,544,834	—	—	—
chr21	35,077,139	24,450,000	24,520,000	70,001
chr22	35,045,071	13,200,000	13,240,000	40,001
chr23	32,480,683	6,270,000	6,690,000	420,001
chr24	31,873,865	22,350,000	22,380,000	30,001
chr25	31,568,348	16,430,000	16,630,000	200,001

### Assessment of the genome quality

To evaluate the accuracy of the final genome assembly, HiFi reads were aligned back to the assembly using minimap2 (version 2.26, https://github.com/lh3/minimap2)^22^. Depth and GC content were calculated in 10-kb windows using SMAtools (version 1.17, http://www.htslib.org/)^23^ and bedtools (version 2.2.29, https://github.com/arq5x/bedtools2)^24^, and a GC-depth scatter plot was generated using R. Assembly completeness was assessed using the BUSCO (version 5.2.2, https://busco.ezlab.org/)^25^ pipeline with the appropriate conserved gene database. In addition, merqury (version 1.3, https://github.com/marbl/merqury)^26^ was used to compute the consensus quality value (QV).

Read mapping rate: A total of 2,369,837 HiFi reads (99.97%) were successfully mapped to the assembly (Table 7).

**Table 7:** The mapping ratio of transcript to the assembled genome of *H. labeo*.

Read Number	Mapped Reads	Percent(%)
2,370,484	2,369,837	99.97

GC-depth plot: No distinct separate clusters were observed in the GC versus coverage depth distribution, indicating the absence of contamination (Fig. 5).

**Fig. 5:**
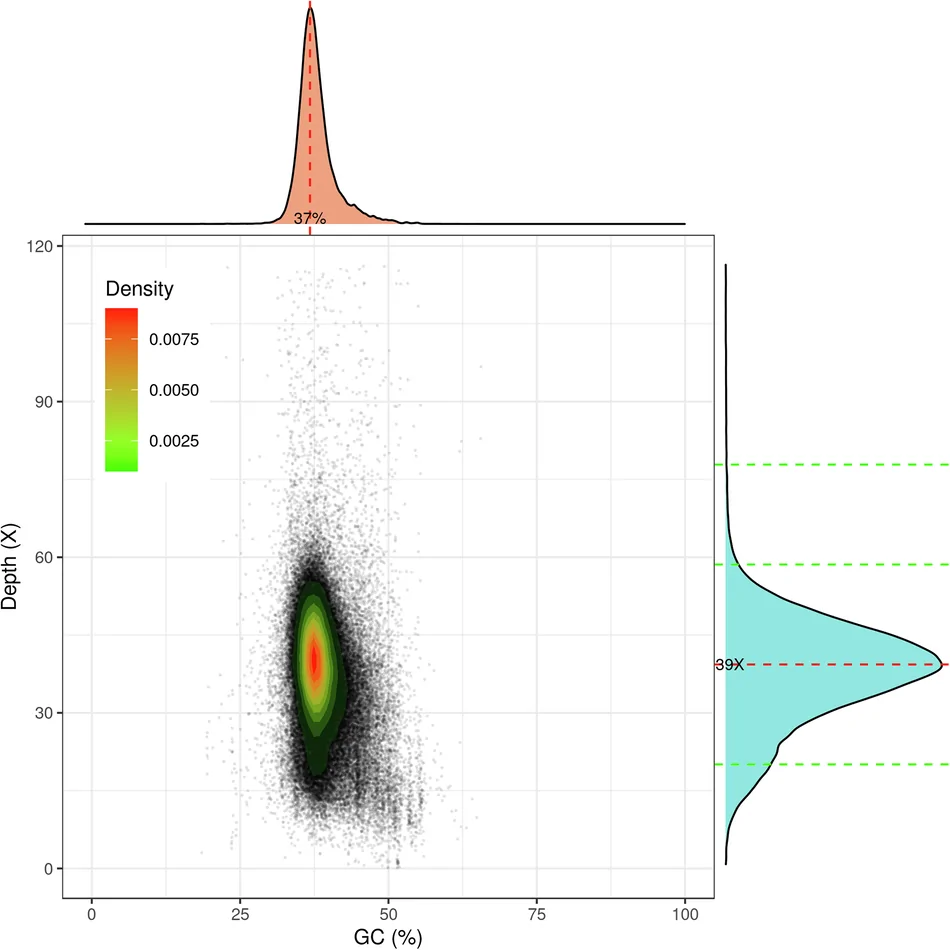
GC-depth distribution chart of *H. labeo*. There are no separate clusters of scattered points in the GC base coverage depth distribution plot.

BUSCO assessment: Out of 7,207 core orthologs, 7,028 (97.5%) were complete (7,000 single-copy [97.1%] and 28 duplicated [0.4%]), 97 (1.3%) were fragmented, and 82 (1.1%) were missing (Table 8).

**Table 8:** Results of the BUSCO assessment for genome assembly in *H. labeo*.

Type	Proteins	Percent(%)
Total BUSCO groups searched	7,207	—
Complete BUSCOs (C)	7,028	97.5
Complete and single-copy BUSCOs (S)	7,000	97.1
Complete and duplicated BUSCOs (D)	28	0.4
Fragmented BUSCOs (F)	97	1.3
Missing BUSCOs (M)	82	1.1

Consensus quality value (QV): The overall genome QV was 61.02; per-chromosome QV values are provided in Table 9 (Table 9).

**Table 9:** The consensus quality value of chromosomes and genomes in *H. labeo*.

Chromosome	QV	Error_Rate	Chromosome	QV	Error_Rate
chr1	64.8928	3.24129e-07	chr14	69.6587	1.08175e-07
chr2	70.6377	8.63429e-08	chr15	68.8623	1.29947e-07
chr3	54.4724	3.57079e-06	chr16	57.402	1.81885e-06
chr4	65.0799	3.10462e-07	chr17	71.5613	6.98030e-08
chr5	57.193	1.90854e-06	chr18	62.995	5.01768e-07
chr6	67.5358	1.76368e-07	chr19	64.4518	3.58774e-07
chr7	66.1955	2.40133e-07	chr20	63.8398	4.13063e-07
chr8	61.5808	6.94903e-07	chr21	64.3427	3.67898e-07
chr9	54.818	3.29764e-06	chr22	66.4413	2.26919e-07
chr10	59.7868	1.05031e-06	chr23	79.8875	1.02625e-08
chr11	63.779	4.18894e-07	chr24	58.5716	1.38943e-06
chr12	63.6121	4.35299e-07	chr25	59.7451	1.06045e-06
chr13	75.225	3.00263e-08	Genome	61.0159	7.91418e-07

### Repeat sequence annotation

A combined strategy using both *de novo* and homology-based approaches was employed for repeat element prediction. For *de novo* annotation, MITE-Hunter^27^ was used to identify MITEs (miniature inverted-repeat transposable elements), which belong to class II transposable elements and are widely distributed in the genome. LTRharvest^28^ and LTR Finder^29^ were used to detect long terminal repeats (LTRs) and LTR retriever^30^ was used to integrate the predictions from both tools. For homology-based annotation, RepeatMasker^31^ was used to search the genome against the RepBase database RepBase (http://www.girinst.org/repbase) to identify known repeat sequences. Combining *de novo* and known repeats, RepeatMasker was applied to mask repetitive regions. Additionally, RepeatModeler^32^ was used to identify other repetitive elements *de novo* from the repeat-masked genome. A total of 595.19 Mb (57.20%) of dispersed repeats were identified, including DNA transposons (28.81%), LINEs (3.35%), SINEs (0.17%), and LTRs (8.75%) (Table 10).

**Table 10:** Statistics of the repetitive elements in *H. labeo* genome.

Type	Number	Length (bp)	Percentage (%)
DNA	1541871	299,798,657	28.81
LINE	56925	20,178,524	1.94
SINE	9929	1,935,418	0.19
LTR	469039	118,292,201	11.37
Satellite	16686	3,708,276	0.36
RC/Helitron	35810	6,403,043	0.62
Retroposon	158	18,637	0.00
Unknown	988176	144,855,662	13.92
Total	3118594	595,190,418	57.20

### Gene prediction and functional annotation

Protein-coding genes were predicted using Augustus (v3.3.2)^33^ and Genscan^34^. Homology-based prediction was performed using tblastn, miniprot (v0.11-r237), and liftoff (v1.6.3)^35^. Protein sequences from closely related species—Da*nio rerio* (GCF_049306965.1), *Ctenopharyngodon idella* (GCF_019924925.1), *Cyprinus carpio* (GCF_018340385.1), *Pseudorasbora parva*(GCF_024679245.1), and *Ictalurus punctatus*(GCF_001660625.3) —were downloaded from NCBI for homology-based evidence. All gene models were merged using MAKER2 (v2.31.10)^36^. Functional annotation was conducted against NCBI NR, Swiss-Prot, KEGG, Gene Ontology (GO), and eggNOG databases^37,38^. A total of 22,518 high-confidence protein-coding gene loci were identified, among which 20,143 (89.5%) were successfully annotated in public functional databases. (Table 11).

**Table 11:** Functional annotation of the predicted protein-coding genes in *H. labeo* genome.

Term	Number	Percentage
Protein-coding genes (unique loci)	22,518	100%
Annotated genes	20,143	89.5%
Annotated by NR	20,012	88.9%
Annotated by SwissProt	15,234	67.7%
Annotated by KEGG	12,687	56.4%
Annotated by GO	11,562	51.4%
Annotated by eggNOG	18,207	80.9%
Not Annotated	2,375	10.5%

### Non-coding RNA prediction and annotation

Non-coding RNA (ncRNA), including rRNA, tRNA, snRNA, miRNA, and snoRNAs, were predicted using INFERNAL (based on Rfam v14.8 and miRBase)^39,40^ and tRNAscan-SE (v1.3.1)^41^. The final annotation identified 2,409 rRNAs, 25,681 tRNAs, 2,704 miRNAs, 2,878 snRNAs, and 396 snoRNAs in the H. labeo genome (Table 12).

**Table 12:** Prediction of Non-coding RNA in *H. labeo*.

Class	Number	Average length(bp)	Total length(bp
rRNA	2,409	157	379,488
tRNA	25,681	74	1,905,923
miRNA	2,704	88	238,587
snRNA	2,878	316	911,463
snoRNA	396	138	54,742

### Quality evaluation of the final assembly

The quality of the final H. labeo genome assembly was evaluated through multiple metrics, including transcript mapping rate, GC-depth distribution, BUSCO completeness scores, QV assessment, and re-mapping of Hi-C data. Collectively, these results confirm the high quality of the assembly. The high contiguity makes it suitable for large-scale genomic analyses. Importantly, the high BUSCO completeness score indicates that nearly all essential eukaryotic genes are present and largely single-copy, a critical requirement for downstream gene annotation and comparative genomics. The consistently high mapping rates observed across different data types (genomic and transcriptomic) not only serve as technical quality indicators but also functionally validate the assembly by demonstrating its ability to accurately represent both raw sequencing reads and expressed gene structures. Thus, this high-quality near-T2T assembly provides a reliable foundation for future studies of gene function, genome evolution, and other downstream analyses.

## Data Records

The raw date of HiFi, Hi-C and ONT sequencing data generated in this study have been deposited in the Sequence Read Archive at NCBI, and the sequencing data are publicly accessible as SRR37458657, SRR37458384, and SRR37469408^42^. RNA-seq data for fin, gil, kidney, liver, spleen and skin tissues have been deposited at the NCBI Sequence Read Archive (SRA) under accessions SRR37469518, SRR37469507, SRR37469516, SRR37469517, SRR37480804, and SRR37481042^42^. The final telomere-to-telomere genome assemblies of *H. labeo* are available in the ENA under BioProject PRJNA1423367 (GCA_056632655.1)^43^. The annotation files of the genome are available at the Mendeley database^44^.

## Technical Validation

The near-complete telomere-to-telomere (T2T) genome assembly of *H. labeo* was validated through multiple approaches. Transcripts mapping ratio showed that a total of 2,369,837 reads were mapped to the genome, with a mapping rate of 99.97%. BUSCO (actinopterygii_odb10) assessment showed that 7,028 (97.5%) of the complete orthologs, indicating high genic completeness. All PacBio HiFi reads (40.36 Gb) using Merqury (k-mer size = 21) were used to evaluate the consensus quality value (QV) of the final genome assembly. The genome-wide QV was calculated to be 61.02, corresponding to an estimated per-base error rate of 7.91 × 10^−7^. GC-depth analysis showed that there are no separate clusters of scattered points in the GC base coverage depth distribution plot, indicating that the sequencing data is free of contamination. These results confirm the high quality and reliability of the assembly.

## Data Availability

The raw data are available at NCBI SRR3745865742, SRR3745838443, and SRR37469408^42^, the genome assembly at Genbank (GCA_056632655.1) and the annotation files at Mendeley Data (https://data.mendeley.com/datasets/4v5pp8xyxg/1).

## Code availability

All software used in this study is in the public domain. Parameter specifications are described in the Methods section, and default parameters were used where no specific settings are indicated. No custom scripts or code were generated during this study.
